# Synthesis of “Dahlia-Like” Hydrophilic Fluorescent Carbon Nanohorn as a Bio-Imaging PROBE

**DOI:** 10.3390/ijms20122977

**Published:** 2019-06-18

**Authors:** Perumalswamy Sekar Parasuraman, Vijaya Rohini Parasuraman, Rajeshkumar Anbazhagan, Hsieh-Chih Tsai, Juin-Yih Lai

**Affiliations:** 1 Graduate Institute of Applied Science and Technology, National Taiwan University of Science and Technology, Taipei 106, Taiwan; parasuuniv@gmail.com (P.S.P.); csvijayarohini@gmail.com (V.R.P.); arrajeshkumar6@gmail.com (R.A.); jylai@cycu.edu.tw (J.-Y.L.); 2 Advanced Membrane Materials Center, National Taiwan University of Science and Technology, Taipei 106, Taiwan; 3 R&D Center for Membrane Technology, Chung Yuan Christian University, Taoyuan 320, Taiwan

**Keywords:** hydrothermal method, carbon nanohorn, bio-imaging

## Abstract

Carbon nanohorns (CNH) were synthesized by a simple conventional hydrothermal method in this study. The CNHs were prepared by the chemical oxidation from the carbonation of Nafion (catalyst) with heparin (carbon resource). The formation of CNH involved two major steps, as described followed. First, the formation of carbon nanorice (CNR) was achieved by carbonation and self-assembly of heparin inside the Nafion structure. Second, the further oxidation of CNR resulted the heterogeneous and porous micelle domains showed at the outer layer of the CNR particles. These porous domains exhibited hydrophobic carbon and resulted self-assembly of the CNR to form the structure of CNHs. The resulting CNHs aggregated into a “dahlia-like” morphology with fluorescence in a diameter of 50–200 nm. The “dahlia-like” CNH showed better fluorescence (450nm) than CNR particles because of the presence of more structural defect. These findings suggest that the hydrophilic fluorescent carbon nanohorns (HFCNHs) synthesized in this study have the potential to be used for in vitro bio-imaging

## 1. Introduction

Carbon nanomaterials are categorized based on their structure as follows: carbon nanotubes (CNTs), graphene, nanodiamonds, carbon nanohorns (CNH), ribbons, onions, hollow carbons nanoparticles, C-dots, etc. [1]. Among these carbon nanomaterials, CNHs are the best candidates for bio-applications owing to their large surface area, which is responsible for their high drug-loading capacity [2]. In addition, the CNH structure is constructed from the mixture of pentagons, hexagons, and heptagons. The carbon atoms in the pentagonal cells are consider to be more chemical reactive sites and can be further functionalized by targeting moiety [3]. Various methods such as the CO_2_ laser ablation of graphite rods [4], arc discharge method [5,6]**,** and Joule’s heating method [7] have been reported for the synthesis of CNHs. However, these methods require very high temperature (>3500 K), which is not desired for the synthesis of CNHs because of their poor solubility in water. Moreover, these methods also require separate chambers, highly stable laser power, and prototype reactors. To overcome these problems, researchers have focused on improving the hydrophilic nature of CNHs and developing new synthetic routes for CNHs under mild conditions. It has been reported that chemical oxidation induces structural defects in CNHs, and the functionalization with carboxylic groups improves their hydrophilic nature [8,9]. The chemical oxidation of CNHs is carried out with HNO_3_ acid [10] or by prolonged heat treatment using O_2_ or CO_2_ [11,12]. The chemical oxidation process produces CNHs with a “dahlia-like” morphology and increased biocompatibility. Moreover, this method facilitates the covalent functionalization of CNH. As a result, chemically oxidized CNHs have gained immense attention as drug carriers and bio-imaging agents [13].

A bio-imaging agent should fulfill the basic requirements of being non-toxic, biocompatible, and soluble in aqueous medium [14]. Fluorescent carbon nanomaterials (FCNs) have emerged as attractive and versatile imaging agents. FCNs are preferred over semiconductor nanocrystals because of the absence of heavy metals and their low cytotoxicity and large binding surface area [15,16]. The fluorescence of carbon nanomaterials can be attributed to the following: the delocalization of π electrons from molecular orbitals, nano-sized/edge state of graphitic domains, and the presence of defect centers (nitrogen vacancy centers) [17]. Even though the aforementioned properties of the fluorescent carbon nanomaterials have disadvantages such as a specific size, chemical functional linker or doping hetero atoms such as N, P, or O allow for an enhanced optical property. In order to overcome these issues**,** in this study, we developed a new route for the synthesis of “dahlia-like“ hydrophilic fluorescent carbon nanohorns (HFCNHs) from Nafion-encapsulated carbon nanorice (NCNR) particles at a mild temperature of 100 °C by a simple hydrothermal chemical oxidation method. The HFCNHs obtained could be used as bio-imaging probes because of the presence of structural defects such as 5-hydroxymethylfurfural (5-HMFs) or other aromatic moieties generated during the NCNR oxidation. The synthesis method developed in this study will pave a new path for the application of HFCNHs as bio-imaging agents and drug carriers.

## 2. Results

### 2.1. Preparation and Morphology Details of Dahlia-Like HFCNH

**Figure 1:**
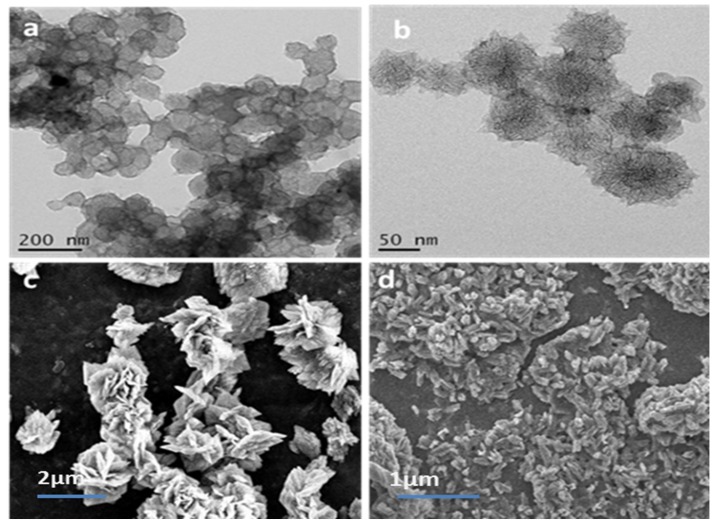
HR-TEM images of HFCNH under (**a**) low and (**b**) high magnification; (**c**) SEM images of HFCNH (1.8mg/mL); (**d**) dissociation of HFCNH in dilute concentration (0.1 mg/mL).

A control experiment demonstrated that separate Nafion and heparin solutions were transparent in appearance and were unaffected by hydrothermal treatment; whereas, a mixture of both turned into formation of Nafion encapsulated carbon nanorice (NCNR) in brownish-black color after hydrothermal treatment by charring of heparin via de-sulfur decomposition. More interestingly, Nafion acted as an assembly template due to its high thermal stability at up to 400 °C and its ionic-cluster-containing heterogeneous porous domain. Furthermore, its swelling property allowed a high degree of morphological freedom and the increase in water volume, facilitating the migration of the water-soluble sodium salt of heparin into the Nafion membrane [18]. Next, the NCNR solution was oxidized with concentrated nitric acid and refluxed for 12 h, after which the solution turned a pale yellow color. “Dahlia-like” HFCNH was examined by high-resolution transmission electron microscopy (HR-TEM). As shown under low magnification (Figure 1a), “dahlia-like” HFCNH assemblies were formed by the aggregation of spherical bundles, which resulted in voids with large interbundle distance [19]. At high magnification (Figure 1b), grown HFCNHs were shown to be typically dahlia- like, with sizes ranging from 50 to 200 nm. Under SEM, a high number of “dahlia-like” aggregates were observed after the chemical oxidation of NCNR (Figure 1c). The HFCNH tended to dissociation when the HFCNH was further diluted with water (Figure 1d). There are two possible pathways for the formation of HFCNH. Firstly, the structural breakdown of NCNR along the curvatures, and secondly, the heterogeneous micelle porous domain of the outer membrane serves as a template for the self-assembly of HFCNH [19]. In addition, the solvation/solvent effect also plays a critical role in the fast proton charge-transfer interaction between the carbon nanomaterial and the nitric acid upon reflux, thereby decreasing the number of voids and providing grown “dahlia-like” HFCNH [20].

### 2.2. Chemical Composition of HFCNH

**Figure 2:**
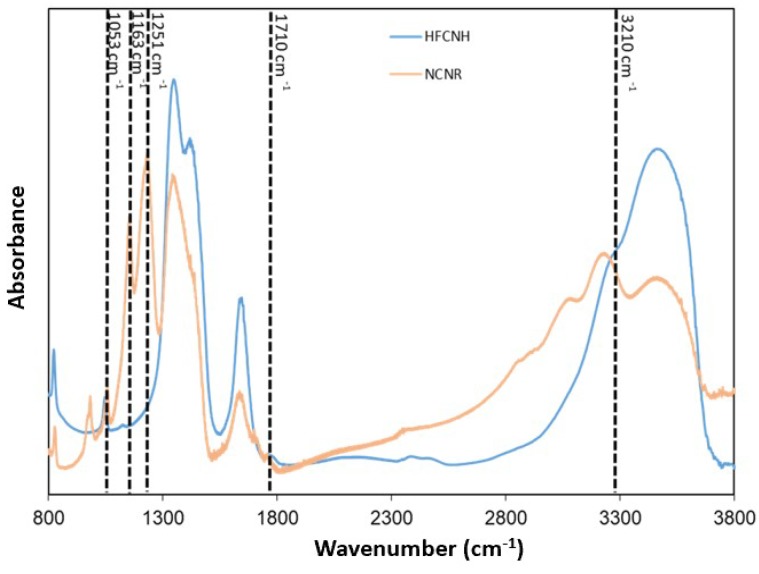
Fourier-transform infrared spectra of NCNR and HFCNH.

For detailed chemical analysis, the functional groups of grown “dahlia-like” HFCNH and NCNR were characterized by FT-IR, as shown in Figure 2. The C–F stretching vibration band of NCNR at 1163 and 1251 cm^−1^ disappeared in the spectrum of HFCNH, indicating the loss of the original Nafion (polytetrafluorethylene backbone). For Nafion, stretching vibrations for SO_3_^−^ and C-F were observed at 1057 and 1173 cm^−1^, respectively [21,22,23]. This result pointed out that the defect of NCNR formed during the nitric acid treatment. In addition, two strong bands at 1710 and 3210 cm^−1^, which can be assigned as C=O and N–H bonds, existed in the HFCNH, which also indicated the formation of oxygen and nitrogen functional group in the HFCNH.

## 3. Discussion.

### 3.1. Elemental Analysis of HFCNH

**Figure 3:**
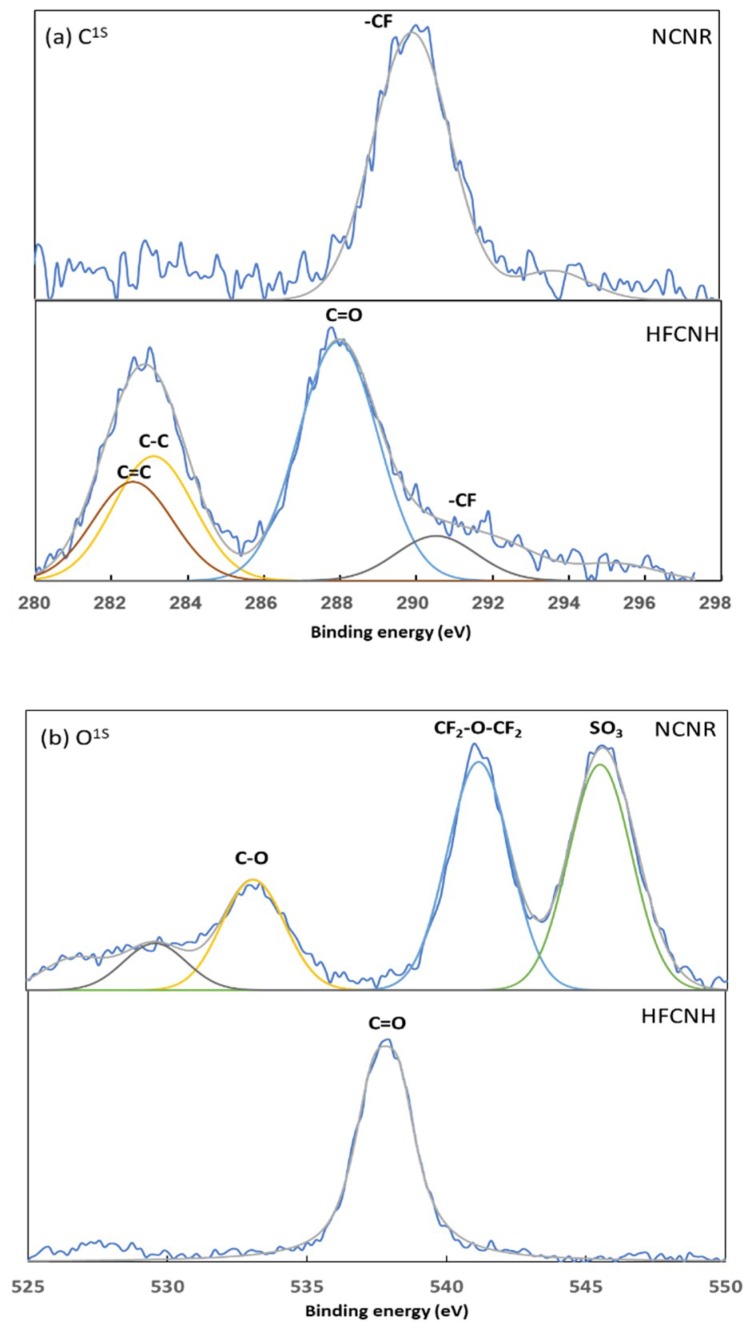
XPS spectra of NCNR and HFCNH with relative peak deconvolutions relative to carbon 1s (**a**) and oxygen 1s (**b**).

Most fluorescent carbon nanomaterials have been characterized with a structure defect on the sp^2^ carbon. Therefore, the chemical structural information of HFCNH has been characterized by XPS, as shown in Figure 3. Chemical oxidation of NCNR resulted in a shift to lower binding energy of carbon, as shown in Figure 3a. For the NCNR, the peak at 289.4 eV, which can be attributed to C–F, might indicate the well coverage of Nafion on the biomass. In addition, the two identical peaks at 282.3 and 287.8 eV in the HFCNH spectrum can be assigned to C=C and C=O, respectively [24,25,26]. Additionally, it was also evident that the sp^2^ carbon atoms displayed a fused aromatic structure (σ*) such as 9-anthracene or other similar organic compounds [27]. As shown in Figure 3b, the peaks at 541 and 546 eV were assigned to S–O and C–O for NCNR. After the chemical oxidation treatment, the binding energy of oxygen shifted to a lower binding energy at 536.8 eV, which implies the presence of C=O. On the other hand, the lack of peaks in the HFCNH F1s spectrum (data not shown here), confirmed the disappearance of Nafion. Oxidizing agents generally attack the ends, sidewalls, and curvatures of graphene. Therefore, we propose that the oxidation of NCNR made the structural defects in the curvatures of NCNR (i.e., outer layer of NCNR), and then those defect NCNR self-assembled to provide “dahlia-like” HFCNHs [28,29].

### 3.2. Absorbance and Fluorescence Properties of HFCNH

**Figure 4:**
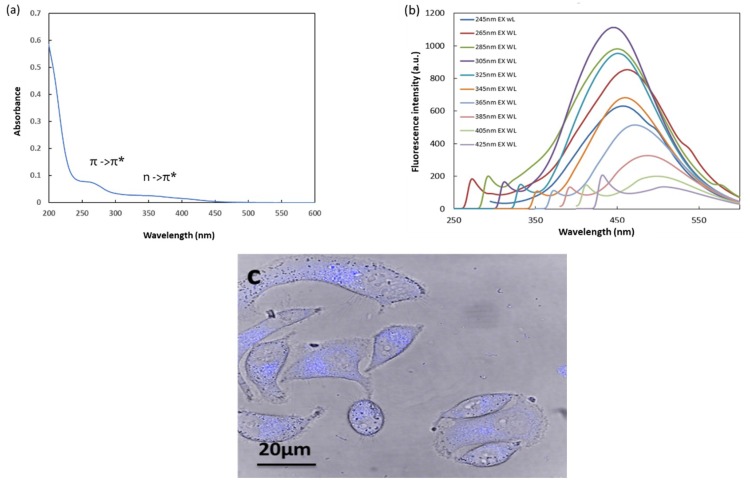
(**a**) UV-Vis absorbance spectrum of HFCNH and (**b**) fluorescence emission spectrum of HFCNH; (**c**) fluorescence image of HFCNH emits blue fluorescence in MDCK cells in vitro.

As shown previously, the maximum absorbance of NCNR is between 275–280 nm due to the presence of π bond-containing carbonaceous structure after hydrothermal treatment [30]. On the other hand, Figure 4a indicates a shift in absorbance to the range of 300–450 nm, confirming the presence of n to π* upon NCNR chemical oxidation. Apparently, HFCNH demonstrated maximum fluorescent emission at 450 nm when excited from 305 nm due to the structure defect or aromatic moieties such as 5-HMF aromatic moiety in NCNR [18]. The emission intensity decreased when the excitation wavelength was increased, as shown in Figure 4b. The fluorescence of HFCNH in the visible region can be attributed to the fluorescent centers produced from defects (oxygen or nitrogen functional groups) in sp^2^ carbon of NCNR during chemical oxidation. This result is similar to that produced by the introduction of the defect structure in the nanodiamonds, resulting in bright fluorescence emission [31,32,33]. It has been proved that the functionalization of carbon materials with polar functional groups not only improves the water solubility, but also makes the energy trapping sites more emissive [34]. As shown in Fig.4c, HFCNH emits blue fluorescence within the nucleus of MDCK cells. Ultimately, these materials can be used as bio-imaging probes for in vitro study.

## 4. Experimental Section

### 4.1. Materials Required

Heparin sodium salt (H3393-100KU Grade-I-A) and Nafion@ perfluorinated resin solution (5% in a mixture of low aliphatic alcohol and 45 wt % water) were purchased from Sigma-Aldrich, St. Loise, MO, USA). Nitric acid 69% was obtained from Baker Co. Ultra-purified distilled water was used in this experiment.

### 4.2. Synthesis of “Dahlia-Like” Hydrophilic Fluorescent Carbon Nanohorn (HFCNH) from Carbon Nanorice (NCNR) via Chemical Oxidation

The synthesis of NCNR was carried out as described in our previous study [18]. In a 50 mL single-necked round-bottom flask containing 3 mL of a NCNR solution, 2 mL of 2N HNO_3_ was added slowly in a closed system, and the mixture was refluxed for 12 h at 100 °C in an oil bath. The brownish NCNR turned pale yellow as “dahlia-like” HFCNH were formed. The supernatant that contained HFCNH was collected and centrifuged at 13,500 rpm for 10 min. Finally, the solid sample of HFCNH was obtained and characterized after the freeze-dried process.

### 4.3. Characterization of HFCNH

Fourier transform infrared (FTIR) spectroscopy of pristine heparin, pre-treated Nafion membrane, NCNR and dahlia-like HFCNH were recorded using a Nicolet 6700 spectrometer (Thermo, Madiason, WI, USA). All materials were well dispersed in distilled water except the Nafion dispersed in methanol to prepare transparent films on CaF_2_ discs. The morphology of “dahila-like” HFNCH was observed through transmission electron microscopy (TEM) images taken by a Hitachi H-7000 transmission electron microscope. HFCNH aqueous solution was characterized by UV-Vis spectroscopy with a JASCO V-670 spectrometer over the range of 200–600 nm using quartz cuvettes. Apparently, fluorescence emission of HFCNH buds were investigated by Spectrofluorometric JASCO FP-8300 with different wavelength ranging from 200–600 nm using fluorescent cuvettes. XPS measurements were performed by a PHI quantera spectrometer with Al l Kα X-ray (1486.7 eV) source and angle of incidence of X-rays ~54.7°. Prior to chamber for XPS measurements, the sample was deposited on silicon substrate and dried in the vacuum oven. Simulation results were deduced from the fits of the core-level lines and analyzed by a set of Lorentzian and Gaussian curves after subtracting the background signal. All of the elements binding energy values were calibrated according to the external standard at Si 2p (99.4 eV).

### 4.4. Fluorescence Cell Image

“Dahila-like” HFCNH internalized by Madin-Darby Canine Kidney cell line (MDCK) was visualized using an LTCS SP5 confocal spectral microscopy imaging system (Leica Microsystems, Wetzlar, Germany). MDCK cells were cultured on cover slides for 24 h and treated with HFCNH. The concentration of HFCNH was fixed at 1.8 mg/mL. After 2 h of incubation, fluorescence was observed by confocal microscopy using 365 nm excitation and a long-pass filter of 450 nm for HFCNH detection.

## 5. Conclusions

**Scheme 1:**
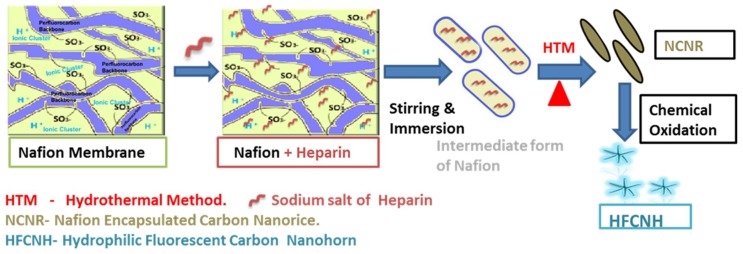
Formation of hydrophilic fluorescent carbon nanohorns (HFCNH) by chemical oxidation of carbon nanorice (NCNR).

In this study, NCNR particles played a vital role in the synthesis of “dahlia-like” HFCNHs through chemical oxidation. Specifically, the geometry of NCNR particles, which is characterized by structural defects and porous domains in the outer layer, directed their breakdown into HFCNHs with a diameter of 50–200 nm. (Scheme 1) The formation of “dahlia-like” HFCNHs can be attributed to their partial hydrophobic nature and aggregation. These HFCNHs can serve as bio-imaging probes for cells owing to their innate fluorescent property. In addition, the horn structure enhances the drug-loading capacity. Thus, this novel material demonstrates significant potential to be used as a guided bio-imaging carrier for hydrophobic anticancer drugs.
